# The Effect of a Unique Region of Parvovirus B19 Capsid Protein VP1 on Endothelial Cells

**DOI:** 10.3390/biom11040606

**Published:** 2021-04-19

**Authors:** Ieva Rinkūnaitė, Egidijus Šimoliūnas, Daiva Bironaitė, Rasa Rutkienė, Virginija Bukelskienė, Rolandas Meškys, Julius Bogomolovas

**Affiliations:** 1 Department of Biological Models, Life Sciences Center, Institute of Biochemistry, Vilnius University, LT-10257 Vilnius, Lithuania; ieva.rinkunaite@bchi.vu.lt (I.R.); egidijus.simoliunas@gmc.vu.lt (E.Š.); virginija.bukelskiene@bchi.vu.lt (V.B.); 2 Department of Regenerative Medicine, Center for Innovative Medicine, State Research Institute, LT-08406 Vilnius, Lithuania; daibironai@gmail.com; 3 Department of Molecular Microbiology and Biotechnology, Life Sciences Center, Institute of Biochemistry, Vilnius University, LT-10257 Vilnius, Lithuania; rasa.rutkiene@bchi.vu.lt (R.R.); rolandas.meskys@bchi.vu.lt (R.M.); 4 Department of Medicine, School of Medicine, UCSD, La Jolla, CA 92093, USA

**Keywords:** parvovirus B19, endothelial cells, VP1u, endothelial stress

## Abstract

Parvovirus B19 (B19V) is a widespread human pathogen possessing a high tropism for erythroid precursor cells. However, the persistence or active replication of B19V in endothelial cells (EC) has been detected in diverse human pathologies. The VP1 unique region (VP1u) of the viral capsid has been reported to act as a major determinant of viral tropism for erythroid precursor cells. Nevertheless, the interaction of VP1u with EC has not been studied. We demonstrate that recombinant VP1u is efficiently internalized by rats’ pulmonary trunk blood vessel-derived EC in vitro compared to the human umbilical vein EC line. The exposure to VP1u was not acutely cytotoxic to either human- or rat-derived ECs, but led to the upregulation of cellular stress signaling-related pathways. Our data suggest that high levels of circulating B19V during acute infection can cause endothelial damage, even without active replication or direct internalization into the cells.

## 1. Introduction

The Human parvovirus B19 (B19V) is a widespread human pathogen that belongs to *Erythroparvovirus* genus of the *Parvoviridae* family. B19V is a small non-enveloped DNA virus, of which the capsid is 20-28 nm in diameter and carries a 5.5 kilobase length genome encoded in a single-stranded DNA molecule [1]. The icosahedral B19V virion consists of two structural proteins—VP1 (83 kDa) and VP2 (58 kDa)—which differ only by the 227 amino acids at the N-terminal region of the VP1-protein, which is called the VP1-unique region (VP1u). Each capsid is composed of 60 capsomers: VP2 is the major capsid protein and constitutes approximately 95% of the total virus particle [2].

B19V infection typically causes erythema infectiosum (also known as the fifth disease), most commonly affecting children [3]. Nevertheless, the age-dependent increase in B19V seropositivity among the general population indicates that B19V actively infects adults [4]. During B19V infection, a drop in reticulocyte numbers and a reduction in hemoglobin is observed [1]. These observations are attributed to the remarkable tropism of B19V to human erythroid progenitor cells (hEPCs) from the bone marrow and liver [5,6,7]. Only a few megakaryocyte–erythroid lineage-derived cell lines are permissive to B19V infection in vitro, with the UT7/Epo-S1 cell line being the most commonly used to study B19V infection [8]. The major factors that determine B19V tropism are erythrocyte P antigen, a glycosphingolipid globoside (Gb4Cer) which has been identified as the primary cellular receptor of B19V [9]. The Gb4Cer-interacting region was identified in the shared C-terminal region of capsid proteins VP1 and VP2 [10]. Ku80 [11] and α5β1 integrin [12] were proposed to act as co-receptors for B19V.

Recent studies have identified the immunodominant VP1u region as a novel determinant of B19V tropism towards erythroid lineage cells, which is required for the virus binding and internalization [13] through the N-terminal 29 amino acids of the VP1u via interaction with a yet-unknown cellular receptor [14]. Furthermore, it has been demonstrated that recombinant VP1u could be used as a model to study B19V internalization [13,14,15]. Nevertheless, despite strictly defined tropism towards hEPCs, the persistence and active replication of B19V have been observed in EC. The B19V genome was found in placental EC and linked to hydrops fetalis [16] in the cardiac endothelium of patients with acute myocarditis or chronic inflammatory cardiomyopathies [17,18,19], or even found in multiorgan endothelium causing endothelialitis [20]. These observations indicate that EC could be a natural target for B19V infection and/or persistence.

The interaction between B19V and EC remains poorly characterized and understood. It was reported that EC in vitro are not permissive for B19V replication, or only a few cells get infected [21]. However, endothelial B19V infection in vitro can be significantly enhanced by the presence of anti-B19V human antibodies, which actively enhance the virus internalization [22]. Moreover, co-infection with adenovirus leads to a substantial augmentation of B19V structural and non-structural proteins in individually infected EC [23]. However, no studies so far have addressed the question of whether VP1u alone can be efficiently internalized by the EC of another species besides the human.

Thus, in this work, we evaluated the effect of the recombinant VP1u region of B19V on EC of different species and origins. We showed that in vitro VP1u can be internalized by rats’ EC of pulmonary trunk blood vessels (PTEC), and is poorly internalized by human umbilical vein endothelial cells (HUVEC). Furthermore, the molecular consequences of VP1u on EC cell cultures were investigated. The expression and phosphorylation of transcription factor activation protein-1 (AP-1) components (c-Fos and c-Jun) and mitogen-activated protein (MAP) kinases in response to recombinant VP1u protein were determined. Our results suggest that, whether it is efficiently internalized into cells or not, VP1u induces a stress signaling cascade, which could lead to endothelial activation.

## 2. Materials and Methods

### 2.1. Cell Cultures

PTECs were isolated from Wistar rats’ pulmonary trunk blood vessels using the outgrowth method [24]. The approval to use laboratory animals for stem cell research was issued by the Lithuanian State Food and Veterinary Service (approval number G2-17, 2014/11/11). The obtained primary cell culture was maintained in Iscove’s Modified Dulbecco’s Medium (IMDM) (Gibco, Carlsbad, CA, USA) supplemented with 10% fetal bovine serum (FBS) (Gibco, Carlsbad, CA, USA) and antibiotics: 100 U/mL penicillin and 100 mg/mL streptomycin (Gibco, Carlsbad, CA, USA). The cells were detached with 0.25% trypsin and 1 mM EDTA mixture in PBS. HUVEC (C-003-5C) (Gibco, Carlsbad, CA, USA), and were cultured on 0.1% gelatin-precoated T-75 culture flasks or well plates in Medium 200 (Gibco, Carlsbad, CA, USA). The media were supplemented with Large Vessel Endothelial Supplement (LVES) (Gibco, Carlsbad, CA, USA) and antibiotics: 100 U/mL penicillin and 100 mg/mL streptomycin. The HUVEC were passaged at ∼70–80% confluence, the monolayer was dissociated with 0.05% trypsin/EDTA (Gibco, Carlsbad, CA, USA), and cells of up to 5–6 passages were used for the experiments. Both cell cultures were maintained at 37 °C in a humidified atmosphere containing 5% CO_2_. 

### 2.2. PTEC Characterization

The PTEC immunophenotyping was performed by flow cytometry. The cells were detached with 1 mM EDTA solution prepared in PBS (Gibco, Carlsbad, CA, USA), and 3 × 10^5^ PTEC were used for each surface marker analysis. The cells were washed twice with 1 % bovine serum albumin (BSA) (Invitrogen, Carlsbad, CA, USA) in PBS and then incubated with primary monoclonal antibodies: CD13, CD14, CD44, CD45, CD54, and CD90 (Table S1, Supplementary Materials) at 4 °C for 30 min. The cells were washed twice with 1% BSA in PBS and then incubated with goat anti-mouse secondary antibody conjugated with R-phycoerythrin fluorescent dye (PA1-84395, Thermo Scientific, Waltham, MA, USA), diluted 1:25 in 1% BSA prepared in PBS at 4 °C for 30 min. The cells were washed twice and analyzed using the BD FACSCanto™ II system (BD Biosciences, San Jose, CA, USA), measuring 10,000 cells. For the determination of the background fluorescence, the PTEC samples were labeled with anti-mouse isotype controls (ab18413, Abcam, Cambridge, UK). The data were analyzed using Flowing Software (version 2.5.1) (Turku Bioscience, Turku, Finland).

### 2.3. Immunocytochemistry

The cells were seeded at a density of 3 × 10^5^ cells/mL and, after 24 h, they were fixed with 4% paraformaldehyde (Carl Roth, GmbH, Germany) in PBS at RT for 10 min, with mild agitation (25 rpm). Then samples were rinsed twice with 0.05% Tween-20 (Sigma-Aldrich, Darmstadt, Germany) in PBS and permeabilized with 0.2% Triton X-100 (Sigma-Aldrich, Darmstadt, Germany) in PBS at RT for 5 min. Next, the cells were blocked with 3% BSA (AppliChem, GmbH, Germany) and 10% FBS prepared in PBS for 30 min. After the blocking, the cells were incubated with primary antibodies (Table S2, Supplementary Materials). All of the primary antibodies were prepared in blocking solution, and the cells were incubated with them at RT for 1 h. Then, the samples were washed three times for 5 min with 0.05% Tween-20 solution, and were incubated with secondary goat anti-mouse Alexa Fluor 488-conjugated antibodies (Invitrogen, Carlsbad, CA, USA) prepared in PBS in the dark at RT for 1 h. After that, the samples were washed three times for 5 min with PBS at RT. Additionally, the cells were stained with 5 µg/mL 4′,6-diamidino-2-phenylindole (DAPI) (Merck Millipore, Burlington, MA, USA) prepared in PBS in the dark at RT for 5 min. Finally, the specimens were washed three times for 5 min with PBS and visualized with a fluorescence microscope (Olympus IX51) (Olympus Europa SE & Co. KG, Hamburg, Germany). The images were processed using ImageJ (1.8.0_112) (National Institutes of Health, Bethesda, MD, USA).

### 2.4. Tube Forming Assay

Matrigel (Geltrex, A1413202, Thermo Fisher Scientific, Waltham, MA, USA) was thawed overnight at 4 °C, and 36 µL was pipetted into 96-well plate. The matrix was gelled by holding the plate at 37 °C for 30 min. After this, 1 × 10^5^ cells/mL PTEC were suspended in serum-free IMDM with antibiotics, and 100 µL of this cell suspension was transferred into the 96-well plates coated with Matrigel. Subsequently, the cells were incubated for 24 h at 37 °C in a humidified 5% CO_2_ atmosphere, and the tube formation process was periodically observed under a light microscope.

### 2.5. Acetylated Low-Density Lipoprotein Uptake

PTEC (7 × 10^5^ cells/mL) was suspended in IMDM growth medium and seeded into the wells of a 6-well plate (3 mL per well); the plates were incubated at 37 °C overnight. The next day, the medium was replaced with a growth medium containing 10–15 µg/mL fluorescently-labeled acetylated low-density lipoprotein (Ac-LDL) (Alexa Fluor^®^ 488 AcLDL, Thermo Fisher Scientific Inc., Waltham, MA, USA), and the cells were incubated at 37 °C for five hours. The uptake of the Ac-LDL by PTEC was evaluated using fluorescence microscopy and flow cytometry.

### 2.6. VP1u Purification

Recombinant VP1u (residues 3–229, UniProtKB/Swiss-Prot: P07299.1) was used in this work. The VP1u and green fluorescent protein-tagged VP1u (GFP-VP1u) were produced in an endotoxin-deficient ClearColi^®^ (Lucigen, Middleton, WI, USA) *E. coli* strain, as described earlier [25]. For the VP1u expression, the pETTrx vector was used. VP1u connected to thioredoxin A (Trx) was affinity-purified from the supernatant on a column containing Ni-NTA-agarose resin. The Trx-VP1u was then cleaved with Tobacco Etch Virus (TEV) protease following the separation of the VP1u from Trx with a second Ni-NTA-agarose resin column. The further purification and concentration of the VP1u were performed on the Phenyl FF column. For the expression of the GFP-VP1u fusion protein, ClearColi^®^ cells were transformed with pETmGFP plasmid containing a VP1u nucleotide sequence (residues 3–229). The GFP-VP1u was purified as VP1u, but after the first Ni^2+^ affinity column, the protein was further purified by gelfiltration on a Superdex^®^ 75 column, and a final polishing step and concentration was performed on a Q Sepharose^®^ column.

### 2.7. Phospholipase A2 Catalytic Activity Assay

The phospholipase enzymatic activity was assessed with a phospholipase A2 (PLA2) assay kit (Cayman Chemical, Ann Arbor, MI, USA). The 1,2-dithio analog of diheptanoylphosphatidylcholine was used as a substrate in this reaction. After thioester’s hydrolysis with PLA2 activity containing protein, the free thiol group at the sn-2 position reacted with 5,5′-dithio-bis-(2-nitrobenzoic acid) and generated a product—5 thio-2-nitrobenzoic acid, which can be detected by measuring the optical density at 412 nm.

### 2.8. Internalization of Recombinant GFP-VP1u

The PTEC and HUVEC (3 × 10^5^ cells/mL) were harvested and incubated with recombinant GFP-VP1u at 4 °C for 1 h. Next, the cells were transferred to a temperature of 37 °C for 1 h, and then washed twice with PBS. The non-internalized GFP-VP1u was removed by trypsinization at 37 °C for 4 min, and then the cells were further washed twice with PBS. The internalized GFP-VP1u was assessed by fluorescence microscopy (Olympus IX51) and flow cytometry (BD FACSCanto II, BD Biosciences, San Jose, CA, USA). GFP was used as the negative control.

### 2.9. Cell Viability Assay

The cell metabolic activity was measured by an MTT (3-(4,5-dimethylthiazolyl-2)-2,5-diphenyltetrazolium bromide) assay. PTEC and HUVEC were seeded at 3 × 10^5^ cells/mL; after 24 h, VP1u at concentrations of 50, 100 and 200 µg/mL was added to fresh media and left to incubate at 37 °C with 5% CO_2_ overnight. After the incubation, the growth media were removed, and the cells were rinsed once with PBS. In total, 0.2 mg/mL MTT (Sigma-Aldrich, Darmstadt, Germany) was dissolved in the media and added to cells, and was then incubated for 1 h at 37 °C. Then, the MTT solution was discarded, and water-insoluble formazan was dissolved in dimethyl sulfoxide (DMSO) (Carl Roth, GmbH, Germany) with gentle shaking at RT for 10 min. The results were quantified using a spectrophotometer Varioskan Flash (Thermo Fisher Scientific, Waltham, MA, USA), reading absorbance at 570 nm.

### 2.10. Real-Time qPCR

Cells were seeded (6 × 10^4^ cells/mL) in 24-well plates, and after 24 hours, at predetermined time points, 100 µg/mL VP1u prepared in fresh media was added. After 1, 2, 4, 6, 12 and 24 h of VP1u exposure, the cells were lysed and the RNA was extracted according to the manufacturer’s protocol using TRIzol Reagent (Invitrogen, Carlsbad, CA, USA). The RNA pellets were dissolved in 0.1 mM EDTA, and their concentrations and purity were determined by a NanoPhotometer P300 (Implen, Inc., Westlake Village, CA, USA) reading at 260 and 280 nm. The RNA’s transcription into cDNA was performed using a High-Capacity cDNA Reverse Transcription Kit (4368814) (Applied Biosystems, Waltham, MA, USA) according to the manufacturer’s instructions. The RT-PCR was performed using the Power SYBR^®^ Green PCR Master Mix (2x) (Thermo Fisher Scientific, Waltham, MA, USA). The total volume of the reaction was 20 µL, and 0.5 µL of the prepared cDNA at the final concentration of 5 ng was used. The selected primers were purchased from Metabion International AG (Planegg/Steinkirchen, Germany), and the final concentration of 200 nM in the reaction was used (Table S3, Supplementary Materials). The mRNAs for the c-Fos and c-Jun expression were quantified by real-time RT-PCR using Bio-Rad CFX96 qPCR system (Bio-rad, Hercules, CA, USA). The initial enzyme activation step started at 95 °C for 10 min. The reaction proceeded with 40 cycles of 95 °C for 15 s and 60 °C for 1 min for gene amplification. The relative gene expression levels were obtained using the ∆∆C_t_ method. The results were normalized to glyceraldehyde-3-phosphate dehydrogenase (GAPDH) transcript and the untreated cell controls.

### 2.11. Western Blot

The cells were lysed in ice-cold lysis buffer (10 mM Tris HCl pH 7.4 (Carl Roth, GmbH, Germany), 50 mM NaCl (Carl Roth, GmbH, Germany), 5 mM EDTA, 50 mM NaF (Sigma-Aldrich, Darmstadt, Germany), 1% Triton X-100 (Sigma-Aldrich, Darmstadt, Germany)) and supplemented with protease and phosphatase inhibitors: aprotinin (1 mg/mL) (Thermo Scientific, Waltham, MA, USA), PMSF (1 mM) (Thermo Scientific, Waltham, MA, USA), and Na_3_VO_4_ (1 mM) (Carl Roth, GmbH, Germany). The protein concentration was estimated by a Bradford assay (Thermo Scientific, Waltham, MA, USA); equal amounts of protein were separated by SDS-PAGE on 12% polyacrylamide gels and transferred onto PVDF membrane (Carl Roth, GmbH, Germany) (semi-dry transfer, 10% methanol). The membranes with transferred proteins were blocked with 5% BSA dissolved in TBST (5 mM Tris HCl pH 7.5, 0.1% Tween 20, 154 mM NaCl (Carl Roth, GmbH, Germany)) for 1 h at room temperature (RT). The primary antibodies diluted in 5% blocking solution were applied at 4 °C overnight (Table S4, Supplementary Materials). After the incubation, the membranes were washed three times for 3–5 min in TBST, and then incubated with secondary antibodies at RT for 1 h. Next, the membranes were washed 3 times for 3–5 min in TBST and incubated with Pierce ECL Western Blotting Substrate (32106, Thermo Scientific, Waltham, MA, USA) for 3 min. The protein expression was visualized in a ChemiDoc XRS+ Molecular Imager (Bio-rad, Hercules, CA, USA) using Image Lab Software. The Western blot images were quantified using the ImageJ program. GAPDH protein was used as a loading control. The levels of the phosphorylated proteins were normalized to those of the total proteins.

### 2.12. Statistical Analysis

All of the experiments were verified by at least three independent experiments. The graphs and statistics were produced using GraphPad Prism version 6.00 (La Jolla, CA, USA). A two-way analysis of variance (ANOVA) followed by Bonferroni’s multiple-comparisons test was performed for the cell viability assay. A one-way ANOVA with Bonferroni’s multiple-comparisons test was used for the RT-qPCR and Western blot data analysis. The data are represented as mean values ± standard deviation (SD). A value of *p* < 0.05 was considered to be statistically significant. The significant differences are marked with symbols and explained below the figures. 

## 3. Results

### 3.1. PTEC Isolation and Characterization

**Figure 1:**
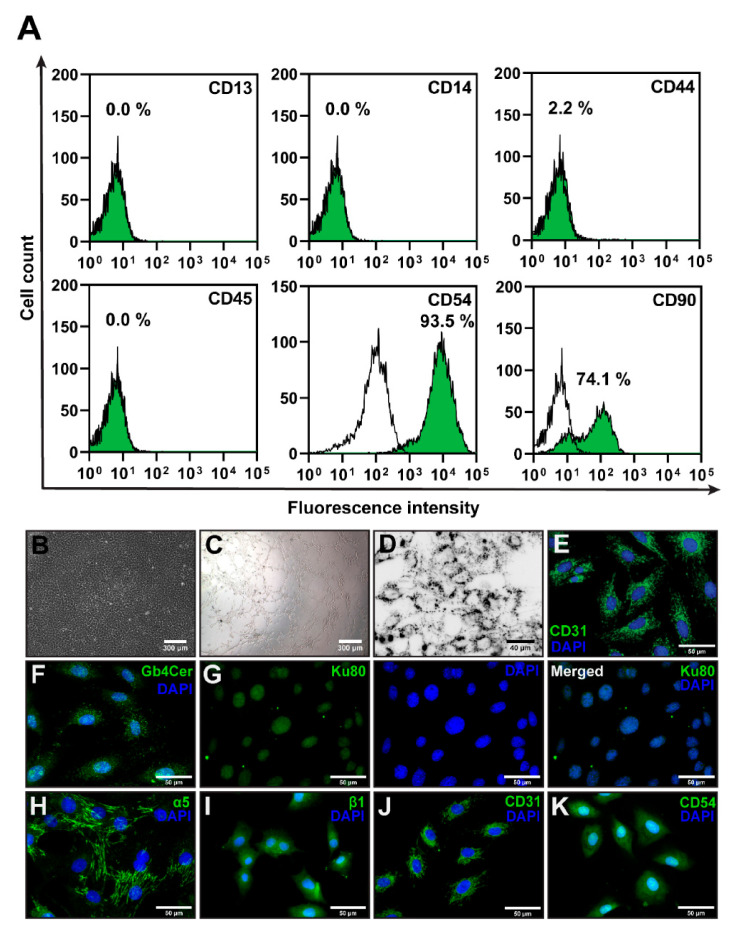
Characterization of the endothelial cells (EC). (**A**) A flow cytometry analysis of the CD13, CD14, CD44, CD45, CD54, CD90 surface markers on EC isolated from Wistar rats’ pulmonary trunk blood vessels (PTEC). White—EC histogram marked with isotopic control, Green—cells labelled with R-phycoerythrin fluorescent dye conjugated antibodies. (**B**) PTEC monolayer. (**C**) PTEC tube-like structures formed on a Matrigel-coated surface. (**D**) The uptake of Alexa Fluor^®^ 488 labeled Ac-LDL by PTEC (Ac-LDL is shown in black). (**E**) An immunofluorescent image of CD31-stained PTECs. (**F**–**I**) The surface expression of primary B19V receptor P antigen (Gb4Cer) and coreceptors Ku80 and α5β1-integrins in PTEC. (**J**,**K**) Human umbilical vein endothelial cells (HUVECs’) surface expression of CD31 and CD54.

Given the close association of various human disorders with the presence of B19V DNA in EC, the objective of our study was to elucidate the effect of B19V-VP1u on EC cells more closely. We chose two EC cultures isolated from rats and humans for our work. We isolated EC from the pulmonary trunk blood vessels of rats (PTEC) (Figure 1B) and characterized the cells by assessing the expression of CD13, CD14, CD44, CD45, CD54 and CD90 surface markers (Figure 1A). The PTECs were positive for CD54 and CD90 surface markers, and were negative for CD13, CD14, CD44, and CD45. The expression of endothelial surface marker CD31 was evaluated using immunofluorescence (Figure 1E). These cells also showed angiogenesis potential (Figure 1C) and were able to uptake acetylated-low density lipoprotein (Figure 1D). Taken together, isolated rat PTECs possess EC characteristics. Moreover, the PTEC cells were positive for B19V receptor P antigen (GB4Cer), and the coreceptors Ku80 and a5b1-integrin (Figure 1F–I). The commercially available HUVEC cell line was used as a human EC system in this study. Periodic examination for CD31 and CD54 surface markers expression (Figure 1J,K) allowed these cells to be used up to six passages without signs of differentiation.

### 3.2. Evaluation of Recombinant VP1u

**Figure 2:**
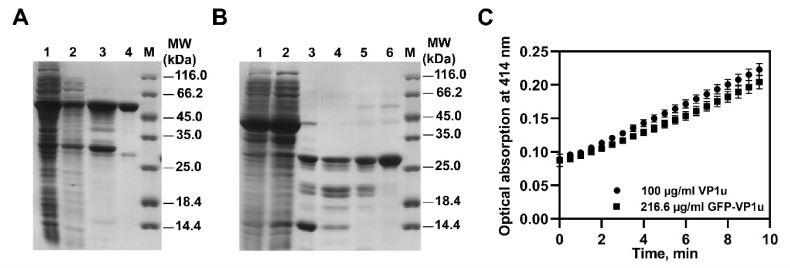
Recombinant VP1us purification and the assessment of the phospholipase A2 (PLA2) enzymatic activity. (**A**) SDS-PAGE gel displaying the GFP-VP1u purification stages: 1—crude extract, 2—cell-free supernatant, 3—proteins eluted from Ni-NTA-agarose column, 4—purified proteins after Phenyl FF column, M—molecular marker. (**B**) SDS-PAGE gel displaying the VP1u purification stages: 1—crude extract, 2—cell-free supernatant, 3—proteins eluted from Ni-NTA-agarose column, 4—separation of VP1u from Trx using a Ni-NTA-agarose resin column, 5—purified proteins after Phenyl FF column, 6—purified and concentrated protein after the additional Phenyl FF column, M—molecular marker. (**C**) PLA2 enzymatic activity results of VP1u and GFP-VP1u recombinant proteins. The results are shown as mean ± SD.

The expression of the B19V-VP1us was carried out in an endotoxin-deficient ClearColi^®^ (Lucigen) *E. coli* strain (Figure 2A,B) (Figure S1, Supplementary Materials). Two high-purity proteins were produced. The predicted molecular masses (from the DNA sequences) corresponded to the SDS-PAGE results: untagged VP1u, 25.3 kDa; GFP-tagged VP1u, 54.8 kDa. The purified proteins also showed PLA2 enzymatic activity (Figure 2C). The measured enzymatic activities of the same molar concentration of VP1u (m = 46 ng; Mr = 25301.99 g/mol) and GFP-VP1u (m = 100 ng; Mr = 54813.24 g/mol) were 29.7 ± 4.1 nmol×min^−1^×mL^−1^ and 26.0 ± 5.2 nmol×min^−1^×mL^−1^ (the specific activities were 2.9 ± 0.4 nmol×min^−1^×mg^−1^ and 6.2 ± 1.2 nmol×min^−1^×mg^−1^), respectively.

### 3.3. Cell Viability after VP1u Exposure

**Figure 3:**
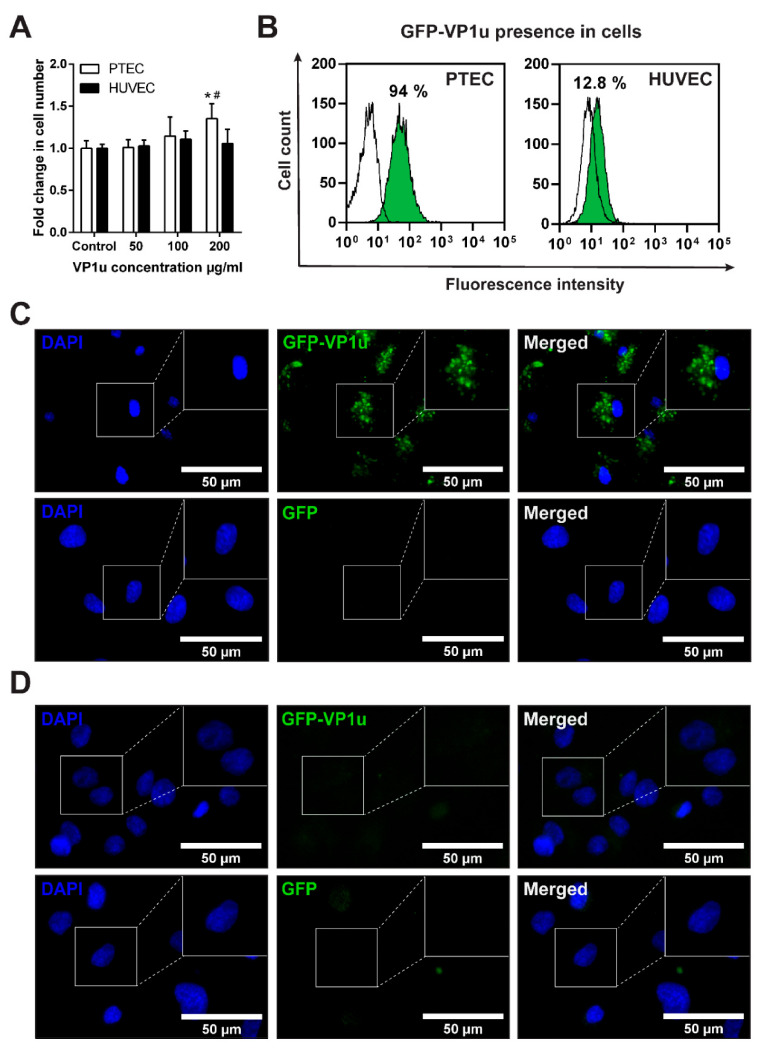
EC viability assay and GFP-VP1u internalization. (**A**) Viability of EC exposed to 50, 100 and 200 µg/mL of VP1u. The data is shown as a ratio of the 24 h control. The data are shown as mean ± SD. *p* ≤ 0.05 (*) compared to the 24 h control; *p* ≤ 0.05 (#) compared to 50 µg/mL concentration. (**B**) Flow cytometry analysis of the GFP-VP1u’s ability to enter the cells. (**C**) GFP-VP1u internalization in PTEC. (**D**) GFP-VP1u internalization in HUVEC.

During B19V infection, up to 10^14^ viral particles/mL can be found in human blood [26,27]. Thus, for the cytotoxicity study, we chose VP1u concentrations corresponding to acute-phase infection levels of B19V particles: 50, 100 and 200 µg/mL of VP1u. Surprisingly, 200 µg/mL of the VP1u increased the PTEC proliferation, whereas HUVEC had no impact on the cell viability (Figure 3A). The increased proliferation potential in PTEC suggested that VP1u could induce stress, which can be followed by the EC activation.

### 3.4. GFP-VP1u Uptake by the ECs

The ECs were exposed to PLA2 enzymatic activity containing GFP-VP1u recombinant fusion protein, and their internalization potential was evaluated (Figure 3B–D) (Figure S2, Supplementary Materials). GFP-VP1u bound to the surface of PTEC and was subsequently endocytosed and dispersed throughout the cytoplasm (Figure 3C). The same uptake process was evaluated by flow cytometry (Figure 3B), and a fluorescence intensity shift of 94% was recorded. The observed uptake of recombinant VP1u is in good agreement with the presence of the known B19V receptors and coreceptors in PTECs, and confirms the functionality of the VP1u preparations. Surprisingly, the uptake of VP1u by non-human primary EC suggests that VP1u is not a determinant of B19V species tropism. In the case of HUVEC, GFP-VP1u was poorly uptaken. Flow cytometry showed a fluorescence intensity shift of just 12.8%, and the GFP-VP1u uptake by HUVECs could not be detected microscopically.

### 3.5. The Effect of VP1u on the Expression and Activation of c-Fos and c-Jun Proto-Oncogenes

**Figure 4:**
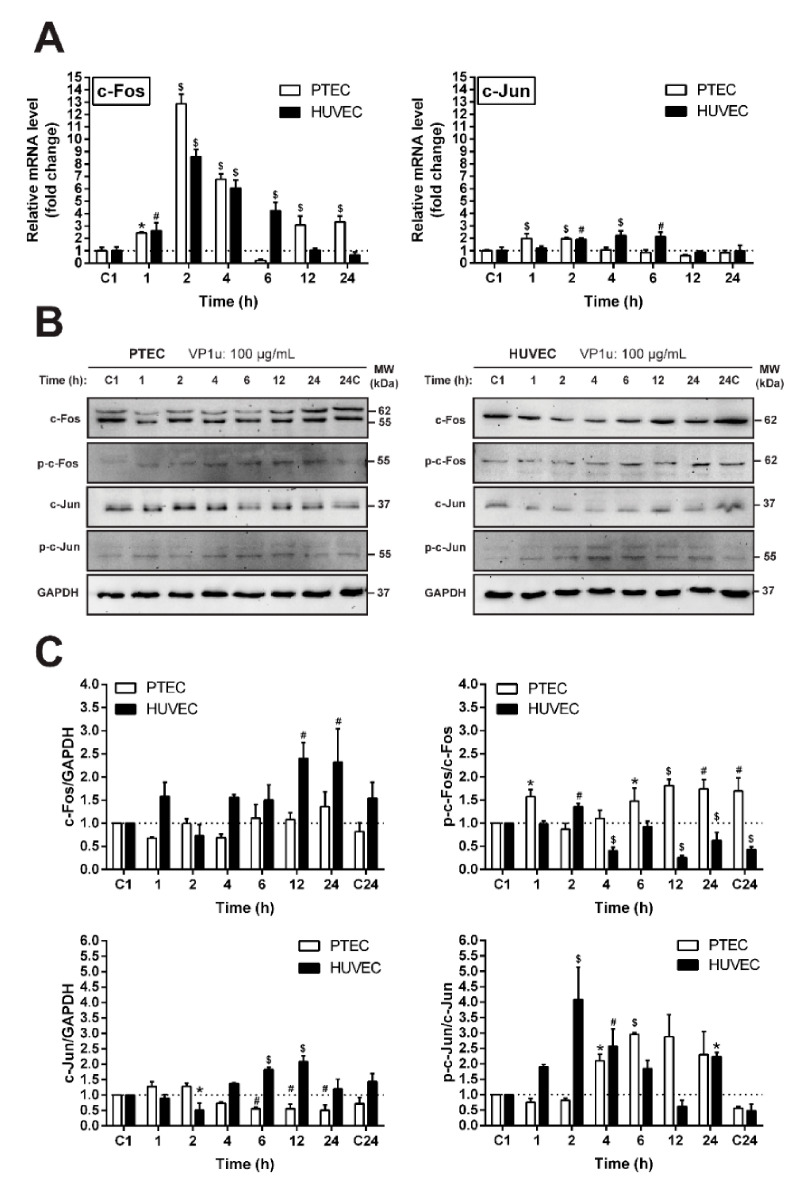
Evaluation of the activation protein-1 (AP-1) components (c-Fos/c-Jun) in EC after exposure to 100 µg/mL VP1u. (**A**) RT-qPCR analysis of the c-Fos and c-Jun mRNA levels in EC at different time points. The data were normalized to GAPDH and presented as a fold change ± SD over the untreated control. (**B**) The abundance of c-Fos and c-Jun proteins in EC. (**C**) Quantification of the total and phosphorylated proteins at different times of VP1u exposure. The results are presented as mean ± SD. *p* ≤ 0.05 (*), *p* ≤ 0.01 (#), *p* ≤ 0.001 ($) vs. control (C1).

In order to assess the molecular consequences of VP1u exposure on ECs, we tracked the changes in the transcription factor AP-1 components c-Jun and c-Fos, which are known to participate in the early cellular events of the stress response (Figure 4A). Our results indicate that VP1u exposure in both studied EC cultures leads to an early upregulation of c-Fos mRNA levels. However, the changes of the c-Jun mRNA levels were not as strongly affected by VP1u exposure. In PTEC, a slight upregulation of the c-Jun transcript levels was observed during the first hours after VP1u exposure, while in the HUVECs, the changes were observed only 2–6 h post exposure compared to the untreated control. The ECs’ exposure to VP1u also induced changes in their c-Fos and c-Jun phosphorylation (Figure 4B,C). The early phosphorylation of the c-Fos protein was detected in both cell lines, while the early increase in c-Jun phosphorylation was more pronounced in HUVECs. Our results indicate that ECs’ exposure to VP1u induces cellular stress, and that the AP-1 complex is likely to participate in these processes.

### 3.6. The Effect of VP1u on the Relative Abundance of Stress Response Proteins

**Figure 5:**
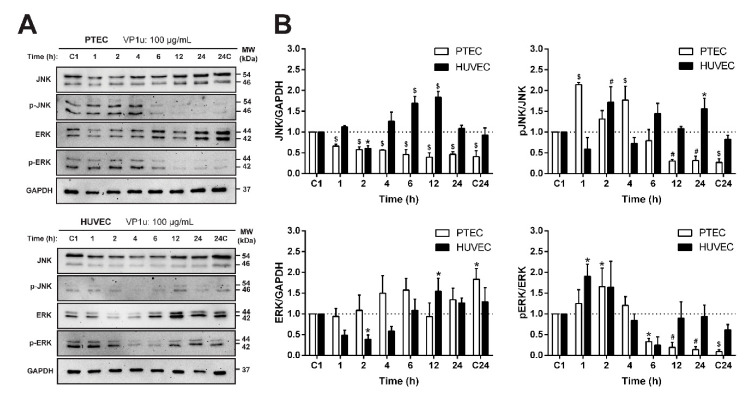
VP1u’s effect on the mitogen activated protein kinases (MAPK) levels and phosphorylation in EC. (**A**) Expression profiles of JNK and ERK proteins after exposing EC to 100 µg/mL of VP1u. (**B**) Quantification of the total and phosphorylated proteins at different times of VP1u exposure. The results are presented as mean ± SD. *p* ≤ 0.05 (*), *p* ≤ 0.01 (#), *p* ≤ 0.001 ($) vs. the control (C1).

PTEC exposed to a 100 µg/mL concentration of VP1u showed increased proliferation potential and changes in the levels of the early response transcription factor AP-1 constituents c-Fos and c-Jun. Usually, the AP-1 complex in cellular stress conditions is activated through the MAP kinases pathway [28]. Thus, we evaluated the expression of the two main kinase families of this pathway: JNK and ERK. The cells were treated with VP1u at a final 100 µg/mL concentration, and the expression kinetics of these proteins were assayed (Figure 5A,B). VP1u affected the kinetics of both the JNK and ERK proteins. The PTEC fraction of the phosphorylated JNK and ERK forms was significantly increased in the early hours after exposure. Meanwhile, in HUVEC, the phosphorylation of JNK was delayed. These findings show that these MAP kinases participate in VP1u-induced stress signaling in both studied EC systems.

Altogether, the mRNA profiling of the early response molecules and the stress-associated protein expression data indicate that both of the studied EC lines respond to the VP1u treatment at a molecular level, although our recombinant VP1u demonstrates different internalization potentials in these cell cultures.

## 4. Discussion

B19V possesses strict species and cell type tropism, efficiently infecting only human erythroid progenitor cells [5,7,9,13,14,15,29]. However, the clinical and experimental data indicate that B19V can infect or persist in EC [16,17,20,30]. The isolated VP1u region of the B19V capsid has been shown to be an adequate model for B19V virus tropism and internalization studies [13,14,15]. However, the interaction of B19V and EC is poorly understood. In this work, we studied the effects of VP1u on EC isolated from rat pulmonary trunk blood vessels (PTEC) and human umbilical vein endothelial cells (HUVEC).

For this study, we produced untagged and GFP-tagged VP1u in the endotoxin-deficient ClearColi^®^ (Lucigen) *E. coli* strain, which ensured that the cellular effects generated by the recombinant protein samples are due to VP1u, and that they are not caused by the lipopolysaccharides (LPS) present in the recombinant protein preparations. As little as 15 pg/mL of LPS can induce significant cytokine production in antigen-presenting cells, such as EC, and recombinant protein samples contaminated with LPS are able to induce cellular activation; thus, the results obtained in this way might be misleading [31,32]. At first, the protein expression was performed in standard BL21 Star™ (DE3) *E. coli* strain; however, the purified proteins had excessive LPS concentrations. Several targeted purification methods were assayed; nevertheless, we were not able to reach acceptably low LPS levels. Thus, this problem was solved using the endotoxin-deficient ClearColi^®^ (Lucigen) *E. coli* strain. VP1u possess PLA2 enzymatic activity, which is important for the virus life cycle [30,33,34]. We used PLA2 enzymatic activity as a readout of our recombinant preparations. Both recombinant proteins, VP1u and GFP-VP1u, showed comparable PLA2 enzymatic activities (VP1u 2.9 ± 0.4 nmol×min^−1^×mg^−1^ and GFP-VP1u 6.2 ± 1.2 nmol×min^−1^×mg^−1^). Our measured B19V PLA2 enzymatic activities of VP1u coincide with other studies [30,33,34].

Next, we evaluated the VP1u internalization potential in rat- and human-derived ECs. PTECs were capable of internalizing VP1u efficiently, whereas HUVECs demonstrated only marginal uptake. Our results agree with previous studies, indicating that HUVECs are poor B19V hosts and deficient for B19V internalization without additional factors [21,22]. We observed that PTECs express all of the key B19V receptors and coreceptors that are also found on HUVECs: Gb4Cer, Ku80, and integrin α5β1 [22,35]. Our results and previous studies suggest that the expression of Gb4Cer, Ku80 and integrin α5β1 alone is not sufficient for efficient B19V internalization [22,36]. It is compelling to speculate that PTEC cells express an as-yet-unknown VP1u receptor, which is not present in HUVECs. Moreover, the efficient VP1u internalization by PTECs suggests that VP1u cannot be considered the sole determinant of cell and species tropism of B19V.

Finally, we investigated the consequences of VP1u exposure in EC. VP1u at concentrations corresponding to clinical viremia levels did not cause cytotoxicity in the studied EC lines, but rather stimulated PTEC proliferation. The increased proliferation could be the result of activated stress signaling pathways [37,38]. We measured the transcript and protein levels of the immediate early genes c-Fos and c-Jun forming the AP-1 transcription factor. AP-1 is a critical mediator of the early response to a variety of stimuli: cytokines, growth factors, stress, and bacterial and viral infections [39,40,41]. AP-1 has been reported to elicit an EC-specific inflammatory profile [42]. Furthermore, the expression of c-Fos and c-Jun can directly cause the activation of human ECs [43], suggesting that these transcription factors mediate the early response to the virus in EC. Exposure to VP1u caused a time-dependent upregulation of c-Fos and c-Jun transcripts, as well as the phosphorylation of c-Fos and c-Jun proteins in both of the studied EC cultures. The activation of mitogen activated protein kinases (MAPK) signaling as a response to virus infection regulates the activity of numerous genes through transcription factors such as c-Fos and c-Jun [28,44,45,46]. Thus, we investigated the phosphorylation levels of two major MAPKs—ERK and JNK—in response to VP1u. We observed the time-dependent increase in phosphorylation of ERK and JNK upon VP1u exposure in both cell cultures. This shows that, upon VP1u exposure, both cell cultures undergo similar cellular stress induction processes independently of their VP1u internalization. Noticeably, the stress response in PTECs was more rapid than that in HUVECs. VP1u possesses PLA2 activity [47,48], which even without internalization could elicit a cellular response [30,49,50]. It was demonstrated that the PLA2 enzymatic activity can modulate the release of arachidonic acid and the precursor of eicosanoids of potent inflammatory mediators [51]. Furthermore, in the clinical trial of the B19V vaccine, the volunteers who received a virus-like particle vaccine possessing PLA2 activity experienced immediate hypersensitivity in the site of the injection. This reaction was linked to the PLA2 activity of the VP1u [52]. Therefore, the PLA2 activity of VP1u could be at least partially responsible for the induction of stress signaling pathways in EC in vitro, and could also be relevant to the pathomechanism of B19V-associated endothelial damage in clinical settings.

Our research confirms that the known viral receptors and coreceptors cannot explain the cellular and species tropism of B19V. More research is needed to determine the exact mechanism of VP1u internalization and the cellular receptors required for it to occur. The current in vitro models used for B19V infection research require specific and complex cellular conditions, which are challenging to implement. We demonstrate that rat PTEC cell culture can be a promising model for further studies of B19V internalization mechanisms.

## 5. Conclusions

We showed that EC from rat pulmonary trunk blood vessels, but not HUVEC, can efficiently internalize recombinant B19V VP1u. VP1u at the concentrations observed during human acute infections was not cytotoxic to EC, but rather induced cellular stress signaling pathways irrespective of the internalization potential. Thus, our data support the hypothesis that high levels of circulating B19V during acute infection in humans can cause endothelial damage even without active replication or internalization in EC.

## Data Availability

The data that support the findings of this study are available from the corresponding author upon reasonable request.

## Supplementary Materials

The following are available online at https://www.mdpi.com/article/10.3390/biom11040606/s1. Figure S1: Chromatograms of GFP-VP1u purification; Figure S2: GFP-VP1u presentence in cells; Figure S3: Images of the original uncropped Western blots used for the preparation of Figure 4; Figure S4: Images of the original uncropped Western blots used for the preparation of Figure 5; Table S1: List of antibodies used for the cell characterization; Table S2: List of the antibodies used for the immunocytochemistry; Table S3: Primer sequences of the genes analyzed by real-time PCR; Table S4: List of the antibodies used in the Western blotting.
