# Supplementary material for: The Effect of a Unique Region of Parvovirus B19 Capsid Protein VP1 on Endothelial Cells
Source: Biomolecules. 2021 Apr 19;11(4):606. doi: 10.3390/biom11040606 (PMC8073096; doi:10.3390/biom11040606)

Table S1: List of antibodies used for cells characterization

Antibody name	Company	Dilution
CD13	Santa Cruz (sc-136484)	1:500
CD14	Merck Millipore (2003608)	1:200
CD44	Cell Signaling (5640S)	1:800
CD45	Merck Millipore (2003607)	1:200
CD54	Merck Millipore (MAB2130)	1:100
CD90	Dianova (T3130)	1:100

Table S2: List of antibodies used for immunocytochemistry

Antibody name	Company	Dilution
Anti-globoside GL4	Matreya LLC (1960)	1:250
Ku80	Cell Signaling (C48E7)	1:200
Anti-Integrin alpha 5	Abcam (ab150361)	1:250
Anti-Integrin beta 1	Abcam (ab179471)	1:250
CD31	Abcam (ab264089)	1:200
CD54	Merck Millipore (MAB2130)	1:250
Alexa Fluor 488	Invitrogen (A-11094)	1:500
Alexa Fluor 488	Invitrogen (A-11001)	1:500

Table S3: Primer sequences of genes analyzed by real-time PCR

Gene	Primers
c-Fos (Rat)	FW: 5′-GGGAGCTGACAGATACGCTC-3′<br>RV: 5′-TCAAGTCCAGGGAGGTCACA-3′
c-Jun (Rat)	FW: 5′-GCCACCGAGACCGTAAAGAA-3′<br>RV: 5′-TAGCACTCGCCCAACTTCAG-3′
GAPDH (Rat)	FW: 5′-AGTGCCAGCCTCGTCTCATA-3′<br>RV: 5′-ATGAAGGGGTCGTTGATGGC-3′
c-Fos (Human)	FW: 5′-GCCTCTCTTACTACCACTCACC-3′<br>RV: 5′-AGATGGCAGTGACCGTGGGAAT-3′
c-Jun (Human)	FW: 5′-CCTTGAAAGCTCAGAACTCGGAG-3′<br>RV: 5′-TGCTGCGTTAGCATGAGTTGGC-3′
GAPDH (Human)	FW: 5′-GTCTCCTCTGACTTCAACAGCG-3′<br>RV: 5′-ACCACCCTGTTGCTGTAGCCAA-3′

Table S4: List of antibodies used in western blotting

Antibody name	Company	Dilution
c-Fos	Thermo Scientific (MA536080)	1:2000
p-c-Fos	Thermo Scientific (PA5-105995)	1:1000
c-Jun	BD Biosciences (610326)	1:500
p-c-Jun	BD Biosciences (558036)	1:500
JNK	Cell Signaling (9258s)	1:1000
p-JNK	Thermo Scientific (700031)	1:1000
ERK	Cell Signaling (137F5)	1:1000
p-ERK	Santa Cruz Biotechnology (sc-16982)	1:500
GAPDH	Invitrogen (MA5-15738)	1:1000
HRP-Goat- anti Rabbit IgG	Life Technologies (656120)	1:10 000
HRP-Goat anti Mouse IgG	Life Technologies (626520)	1:10 000

Figure S1: Chromatograms of GFP-VP1u purification. A - Chromatogram of GFP-VP1u purification in a Q sepharose column. Black line – optical absorption at 280 nm, red – conductivity. B – Chromatogram of GFP-VP1u purification in a Superdex75 column.

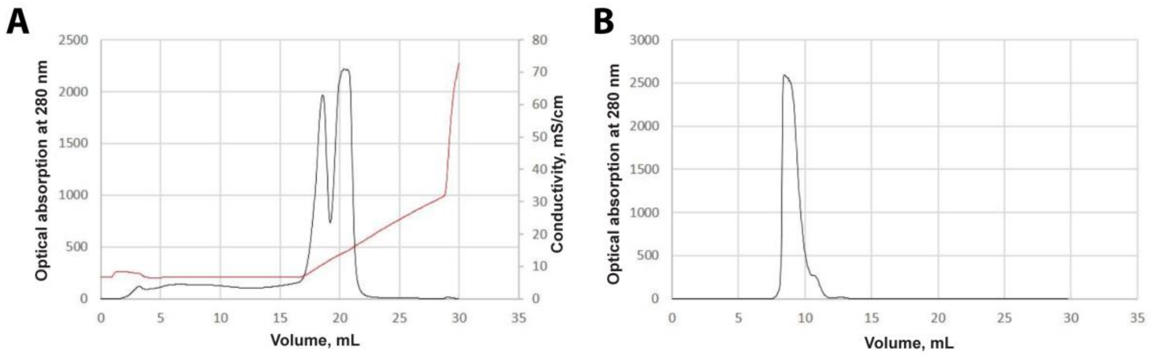

Figure S2: **GFP-VP1u presentence in cells.** GFP was used as the negative control.

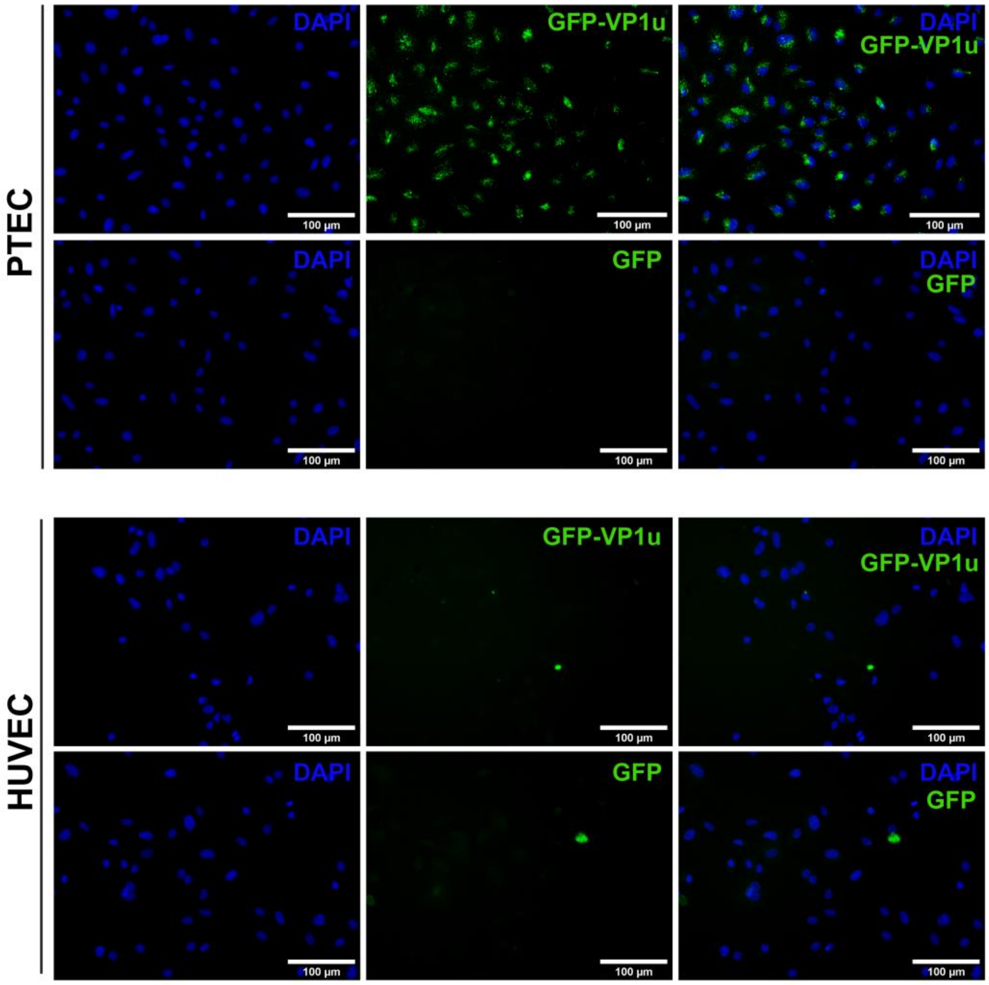

Figure S3: Images of original uncropped Western blots used for the preparation of Figure 4

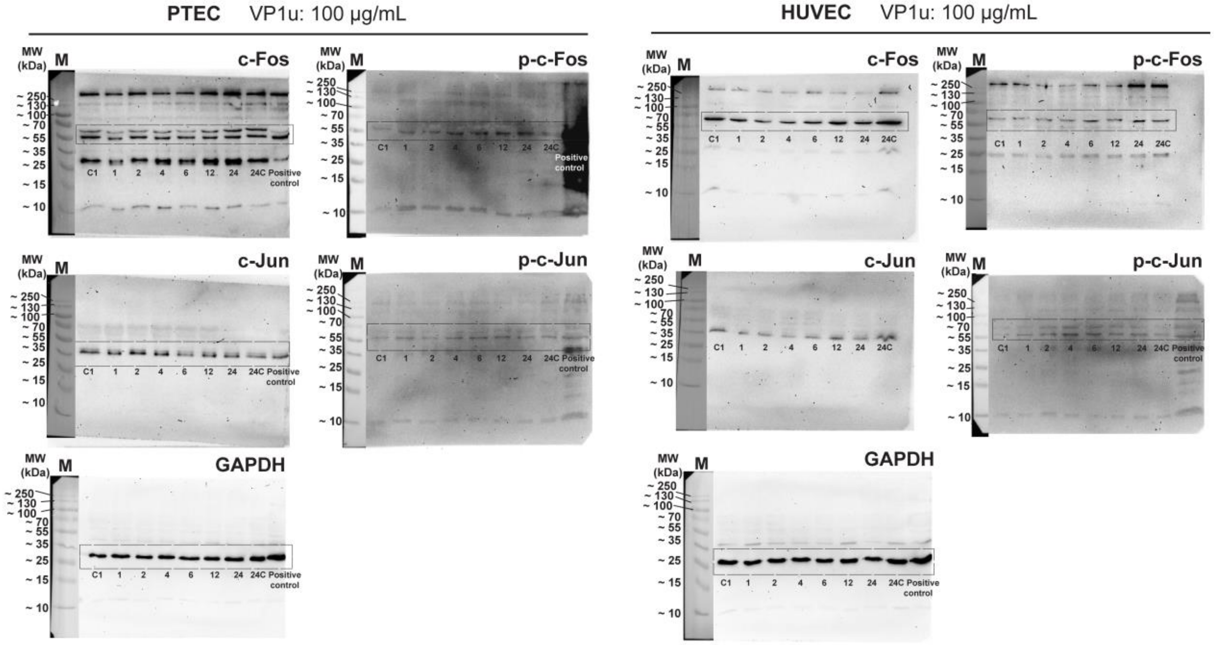

Figure S4: Images of original uncropped Western blots used for the preparation of Figure 5

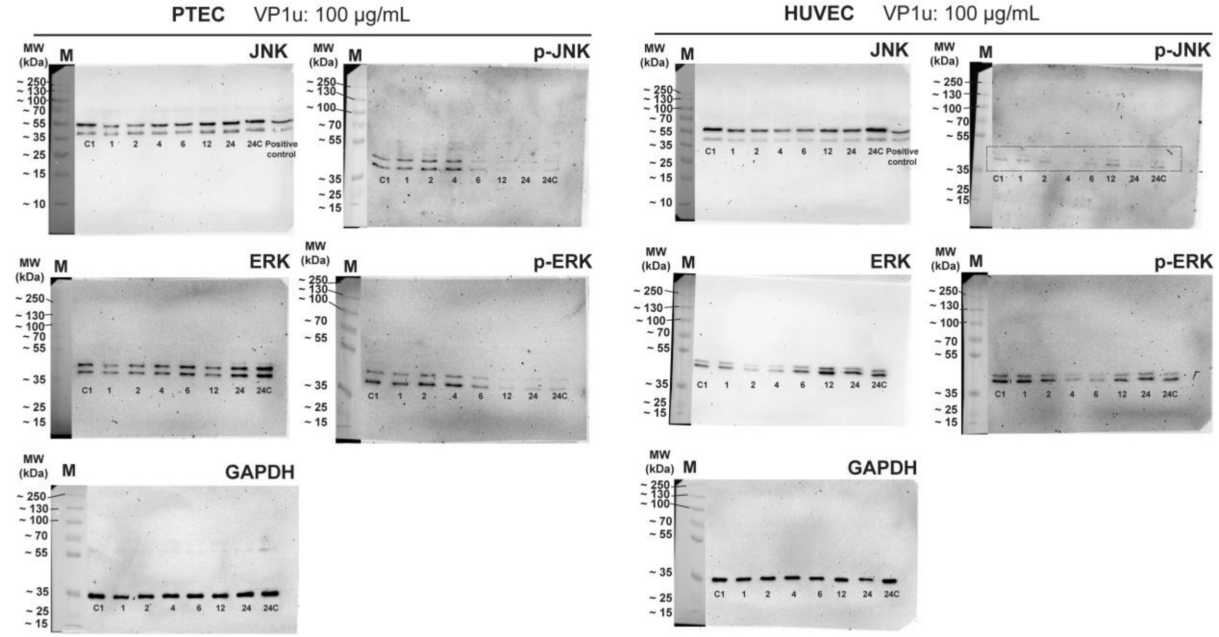

Supplement: Supplementary file 1 [file biomolecules-11-00606-s001.zip › biomolecules-1172491-supplementary.pdf]
